# Supplementary figures and images for: Optimizing speleological monitoring efforts: insights from long-term data for tropical iron caves
Source: PeerJ. 2021 Apr 16;9:e11271. doi: 10.7717/peerj.11271 (PMC8054738; doi:10.7717/peerj.11271)

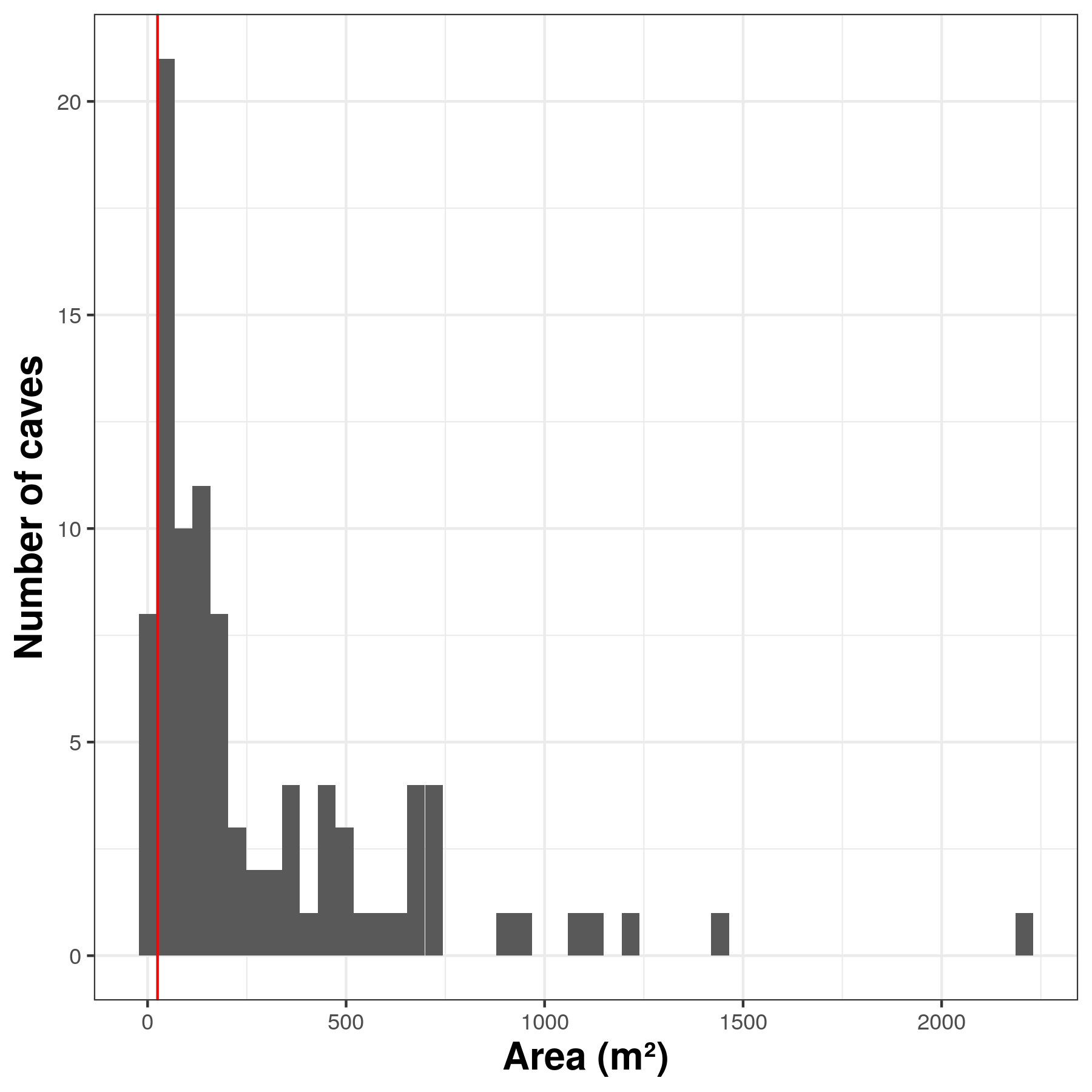

Supplement: Supplemental Information 2 — The red vertical line indicates an area of 25 m² (5x5m caves). [file peerj-09-11271-s002.png]
