# Supplementary material for: Optimizing speleological monitoring efforts: insights from long-term data for tropical iron caves
Source: PeerJ. 2021 Apr 16;9:e11271. doi: 10.7717/peerj.11271 (PMC8054738; doi:10.7717/peerj.11271)
Supplement: Supplementary file 3 [file peerj-09-11271-s001.odt]

Supplemental Files

Table S1: Environmental and landscape metrics assessed.

| Metric | Description | Calculation |
| --- | --- | --- |
| Landscape metrics |  |
| Distance to mine | Topographic distance to the closest open-pit mine, calculated for years 2015-2019 | TopoDist package |
| Forest cover | Percentage forest cover in buffer areas of 500 and 1000m, calculated for years 2015-2019 | landscapemetrics package |
| Canga cover | Percentage Canga cover in buffer areas of 500 and 1000m, calculated for years 2015-2019 | landscapemetrics package |
| Mining cover | Percentage mining cover in buffer areas of 500 and 1000m, calculated for years 2015-2019 | landscapemetrics package |
| Vegetation cover | Percentage of natural vegetation cover in buffer areas of 500 and 1000m, calculated for years 2015-2019 | landscapemetrics package |
| Environmental metrics |  |
| Area | Area in square meters of the accessible space of a cavity | Field measurements |
| Dev Rainfall | Deviation in average bimonthly rainfall in relation to the historical expectancy (20 years) | Field measurements |
| Temperature | Mean internal temperature (ºC) on the date of the surveys | Field measurements |
